# PrEP Disparities Among Transgender Feminine, Transgender Masculine, Nonbinary, and Gender Expansive Youth and Young Adults in the United States

**DOI:** 10.1007/s10461-024-04590-x

**Published:** 2025-01-31

**Authors:** Anne E. Fehrenbacher, Demetria Cain, Joshua A. Rusow, Swetha Lakshmanan, Dianna Polanco, Demi Ward, Yara Tapia, Risa P. Flynn, Patrick S. Sullivan, W. Scott Comulada, Keith J. Horvath, Cathy J. Reback, Dallas T. Swendeman, Adolescent Medicine Trials Network for HIV/AIDS Interventions (ATN) CARES Team, Mary Jane Rotheram-Borus, Sue Ellen Abdalian, M. Isabel Fernandez, Jeffrey D. Klausner, Sung-Jae Lee, Maryann Koussa, E. E. Weiss, Ronald Brookmeyer, Wenze Tang, Karin Nielsen, Yvonne Bryson, Tara Kerin, Chelsea Shannon, Ruth Cortado, Kate Mitchell, Elizabeth Mayfield Arnold, Norweeta Milburn, Marguerita Lightfoot, Danielle Harris, Jasmine Fournier

**Affiliations:** 1 Department of Population and Public Health Sciences, Keck School of Medicine, University of Southern California, Los Angeles, CA USA; 2 Department of Psychology, Hunter College, City University of New York, New York, NY USA; 3 George Warren Brown School of Social Work, Washington University, St. Louis, MO USA; 4 Friends Research Institute, Inc, Los Angeles, CA USA; 5 Anderson School of Management, University of California, Los Angeles, CA USA; 6 McKinsey & Company, New York, NY USA; 7 Medical College of Wisconsin, Milwaukee, WI USA; 8 Department of Musicology and Ethnomusicology, Tulane University, New Orleans, LA USA; 9 Transgender Caucus, Los Angeles County Commission on HIV, Los Angeles, CA USA; 10 Los Angeles LGBT Center, Los Angeles, CA USA; 11 Department of Epidemiology, Rollins School of Public Health, Emory University, Atlanta, GA USA; 12 Department of Psychiatry and Biobehavioral Sciences, Semel Institute for Neuroscience and Human Behavior, University of California, Los Angeles, CA USA; 13 UCLA Center for HIV Identification, Prevention, and Treatment Services, University of California, Los Angeles, CA USA; 14 Department of Psychology, San Diego State University, San Diego, CA USA

**Keywords:** Transgender, Adolescents, PrEP, HIV prevention, Disparities

## Abstract

This study assessed disparities in pre-exposure prophylaxis (PrEP) use among transgender and gender expansive youth and young adults (N = 477) between 15 and 24 years old in the CARES (ATN 149) and TechStep (ATN 160) study protocols within the National Adolescent Medicine Trials Network for HIV/AIDS Interventions (ATN). Structural equation modeling was used to test mediation pathways between gender identity and PrEP uptake among the full sample and stratified by sex assigned at birth. Lifetime PrEP uptake was higher among those assigned male at birth (26%) versus assigned female at birth (9%), explained by greater structural and behavioral risks and perceived need for PrEP, especially among trans women. Among those assigned female at birth, PrEP uptake was higher among trans men (12%) than nonbinary participants (6%). Our findings characterize key structural and behavioral drivers of PrEP use and highlight the need to reduce barriers to healthcare for trans youth, particularly in the South.

## Resumen

Este estudio evaluó las disparidades en el uso de profilaxis previa a la exposición (PrEP) entre jóvenes y adultos jóvenes transgénero y de género expansivo (N = 477) entre 15 y 24 años en los protocolos de estudio CARES (ATN 149) y TechStep (ATN 160) dentro del Red Nacional de Ensayos de Medicina en Adolescentes para Intervenciones contra el VIH/SIDA (ATN). Se utilizó un modelo de ecuaciones estructurales para probar las vías de mediación entre la identidad de género y la ingesta de PrEP entre la muestra completa y estratificada por sexo asignado al nacer. La ingesta de PrEP durante toda la vida fue mayor entre los hombres asignados al nacer (26%) versus las mujeres asignadas al nacer (9%), lo que se explica por mayores riesgos estructurales y conductuales y la necesidad percibida de PrEP, especialmente entre las mujeres trans. Entre los asignados como mujeres al nacer, la utilización de PrEP fue mayor entre los hombres trans (12%) que entre los participantes no binarios (6%). Nuestros hallazgos caracterizan los impulsores estructurales y conductuales clave del uso de PrEP y resaltan la necesidad de reducir las barreras a la atención médica para los jóvenes trans, particularmente en el Sur.

## Introduction

Transgender and gender expansive individuals are those whose gender identity differs from, or expands beyond, the sex assigned to them at birth [1]. Transgender (hereafter: trans) adults are 32 times more likely to acquire HIV than their cisgender peers [2–5]. Although trans and gender expansive youth and young adults (TGEYYA) are disproportionately affected by HIV and other health disparities, they are understudied and underserved [6–12]. Furthermore, most HIV prevention research with TGEYYA has focused on trans women, with few studies investigating the unique life circumstances and needs among subgroups of trans adolescents [13, 14]. A recent scoping review on determinants of pre-exposure prophylaxis (PrEP) implementation for HIV prevention with trans populations [15] identified only four peer-reviewed manuscripts on PrEP studies that explicitly included trans men–of which two had samples sizes of trans men too small to evaluate their barriers and facilitators to PrEP uptake [16, 17]–and only one PrEP study that explicitly included nonbinary participants [18].

There are many challenges in evaluating HIV prevention research among TGEYYA, such as inconsistent language regarding the population, flawed or nonexistent population-based estimates, and inattention to or exclusion of trans men and nonbinary individuals who are often mistakenly classified as low risk for HIV acquisition [9, 19–22]. Estimates published in 2022 suggest that there were more than 1.6 million trans individuals over age 13 in the United States [21]. Furthermore, young adults ages 18–24 years old are more likely to identify as trans or nonbinary than other age groups [23, 24].

TGEYYA endure multiple intersecting forms of marginalization that contribute to mutually reinforcing structural and behavioral syndemics, which cumulatively heighten susceptibility to HIV infection [25]. Violence and social stigma against TGEYYA often start early and extend across the life course [26]. Frequent family rejection can lead to cycles of housing instability, food insecurity, and poverty, amplified by institutional discrimination in education and employment, which can lead to a reliance on informal work, such as exchange sex to earn money [27–30]. It is estimated that 1 in 2 young trans women and 1 in 3 young trans men report exchanging sex for money or other needs, such as a place to stay [31–34]. TGEYYA are frequently profiled by law enforcement for their gender expression and incarcerated at higher rates than cisgender peers, exacerbating mental health and substance use disparities [35–37]. Approximately 1 in 5 trans people in the U.S. have spent time in prison [38]. Substance use is 2.5 to 4 times higher and polysubstance use is 4 times higher among TGEYYA compared to cisgender adolescents [39]. Few studies have evaluated differences in structural and behavioral risks among TGEYYA to determine which groups are most susceptible to HIV and in need of preventive interventions, such as PrEP [10, 15, 40].

TGEYYA may be reticent to seek out HIV prevention services because of a history of discrimination, stigmatization, and pathologizing by health professionals. Indeed, many TGEYYA do not share their gender identity or sexual behaviors with healthcare providers out of fear of discrimination or being outed to others [10, 41–43]. Among trans adults, lower rates of healthcare utilization have been documented due to anticipated discrimination, and while not assessed among youth, TGEYYA must also consider privacy concerns if they receive health insurance through a parent [30, 44, 45]. These barriers to healthcare might limit TGEYYA’s perceived need for PrEP as a tool for HIV prevention, and impair their ability to move through the PrEP cascade and HIV prevention continuum from no engagement, to HIV testing, to linkage to care, and finally to retention in routine healthcare and HIV prevention services [40, 46–51].

HIV prevention research with TGEYYA has almost exclusively focused on trans women, and less is known about risk and protective behaviors among trans men, trans masculine individuals, and nonbinary adolescents, irrespective of sex assigned at birth [15]. Over a decade of research has reliably estimated the prevalence of HIV among primarily adult trans women in the U.S. to be at least 1 in 5 [4, 52]. Although national estimates among trans masculine and nonbinary populations are not available, a few small non-probability samples of primarily adult trans men have demonstrated rates of HIV from 2 to 10%, much higher than the national HIV prevalence of 0.4% in the U.S. [5, 12, 53, 54].

Trans masculine and nonbinary individuals assigned female at birth who have sex with men are at increased risk for HIV transmission if they have condomless receptive anal or vaginal sex while not on pre-exposure prophylaxis (PrEP). Furthermore, transmission risk is elevated among those taking testosterone, which can cause vaginal atrophy and decreased lubrication [55–58]. Nonetheless, trans masculine individuals have been excluded from all PrEP trials despite high rates of eligibility for PrEP, and gender expansive and nonbinary populations assigned female at birth have been largely invisible in HIV research [13, 59–61]. Studies that informed CDC clinical guidelines on PrEP included very small numbers of trans women and no trans men; as such, efficacy data relevant for trans masculine and nonbinary populations are not available, and PrEP remains underutilized among all subgroups of TGEYYA [14, 47, 62, 63].

Additionally, previous studies have shown that while TGEYYA have significant needs for PrEP, uptake is differentially associated with both sex assigned at birth and gender identity, as well as access to healthcare [64]. Trans women tend to have high perceived need for PrEP aligned with their self-reported sexual behaviors, higher rates of HIV testing and positivity for HIV and STIs than other TGEYYA subgroups [65], and also higher PrEP awareness and uptake than other TGEYYA subgroups [66], yet they still face barriers such as stigma, medical mistrust, affordability, and accessibility [41, 43, 67]. Trans men report less exposure to targeted PrEP outreach, which affects their uptake and perceived need for PrEP. Nonbinary individuals tend to face greater barriers to care generally than trans women and trans men related to less visibility and provider knowledge of their identities and sexual behaviors, and lack of inclusive interventions to meet their needs [68–71].

The current analysis aimed to address gaps in the literature on PrEP use among TGEYYA, attending to differences by gender identity and sex assigned at birth. The objectives of the current analysis were to assess disparities in current and lifetime PrEP uptake among TGEYYA and potential mechanisms that might explain these disparities. In our investigation of mechanisms, we considered mediation by structural risk factors (housing instability, incarceration, low income, unemployment, and uninsurance), behavioral risk factors (condomless sex, multiple sex partners, exchange sex, intimate partner violence, and polysubstance use), perceived need for PrEP, and healthcare utilization based on the participant’s self-reported history of HIV testing, linkage to care, and retention in routine health care. We hypothesized that increased structural and behavioral risk factors would be associated with greater perceived need for PrEP, more healthcare utilization, and ultimately PrEP uptake.

## Methods

The 2017–2022 cycle of the National Adolescent Medicine Trials Network for HIV/AIDS Interventions (ATN) consisted of three research networks (or U19s), and each U19 consisted of several study protocols. This cross-network analysis used baseline data from a pooled sample of TGEYYA participants (N = 477) in the CARES and TechStep study protocols within the ATN.

### Pooled Study Sample and Inclusion Criteria

Participants who met the following criteria were included in the pooled sample for this cross-network analysis: 1) 12–24 years old at study enrollment; 2) seronegative for HIV infection at study enrollment; 3) gender identity different from sex assigned at birth; and 4) enrolled in either CARES (n = 197) or TechStep (n = 284) parent studies. Four participants were excluded due to missing information for sex assigned at birth. The final pooled sample included 477 TGEYYA participants. An overview of the CARES and TechStep parent study designs, eligibility criteria, participants, and procedures are provided below.

### Parent Study Data and Samples

#### CARES Study Design

Participants for this cross-network analysis were drawn from CARES ATN Protocol 149, which enrolled seronegative youth into a four-condition randomized controlled trial for technology-based interventions to promote completion of each step of the HIV prevention continuum among youth at high risk for HIV [72].

#### CARES Participants

From May 2017 to August 2019, 1,482 seronegative participants between the ages of 12–24 years old were enrolled in ATN 149. Inclusion criteria were: (1) a negative rapid HIV result on a 4th generation test at screening (Alere, Waltham, Massachusetts) AND (2) self-reporting at least three HIV-related structural or behavioral risks among the following: past 12-month history of condomless anal sex, an HIV-positive sex partner, sharing needles for injecting drugs, illicit substance use (excluding marijuana), or a positive STI test; having ever been homeless, hospitalized for a mental health disorder, or been to jail or on probation in their lifetime; OR (3) identifying as trans or nonbinary/gender expansive – irrespective of sex assigned at birth – or as gay, bisexual, or other men who have sex with men (GBMSM); (4) able to read and speak English; and 5) age 12–24 years. Adolescents who self-identified as sexual or gender minority were weighted more heavily in eligibility scoring due to their higher representation in the domestic HIV epidemic. Baseline data from seronegative CARES participants who selected a gender identity different from their sex assigned at birth were included in this cross-network analysis (n = 197). These TGEYYA constituted 13% of the total 1,482 participants in ATN 149.

#### CARES Procedures

CARES was conducted in Los Angeles and New Orleans. Youth were recruited via: (1) community-based organizations (CBOs) and clinics that served gay, bisexual, and trans youth; homeless youth; and youth on probation or released from incarceration; (2) dating apps (e.g., Jack’d and Scruff); (3) community-based events (e.g., PRIDE, Teen PRIDE); and (4) referrals from other enrolled study participants [73]. Following consent, participants completed a tracking form and baseline assessment verbally administered by interviewers, and rapid diagnostic tests for HIV, STIs (syphilis, chlamydia, and gonorrhea), and substance use (alcohol, marijuana, opiates, methamphetamine, cocaine, and benzodiazepines). The study was approved by the Institutional Review Board at the University of California, Los Angeles, which served as the IRB of Record for all participating institutions.

#### TechStep Study Design

TechStep was Protocol ATN 160 within the University of North Carolina/Emory Center for Innovative Technology (iTech) within the Adolescent Medicine Trials Network for HIV/AIDS Interventions (ATN) [74]. The TechStep design included a three-condition, technology-based, randomized controlled trial, with a stepped care approach, for reducing sexual risk behaviors and increasing PrEP initiation among HIV-negative trans youth and young adults.

#### TechStep Participants

From October 2019 to September 2021, 284 participants completed the TechStep baseline Audio Computer Assisted Self Interview (ACASI) assessment. Inclusion criteria were: (1) self-identify as trans feminine, trans masculine, gender expansive, or any term that reflected incongruity between sex assigned at birth and current gender identity; (2) aged 15–24 years old; (3) self-report vaginal or anal sex (either insertive or receptive) with another person in the last 12 months; (4) verified HIV negative serostatus; (5) a mobile device with SMS and Internet access capabilities; and (6) able to read and speak English (as the interventions were built in English). Individuals were excluded if they did not meet all eligibility criteria. All 284 participants who completed the baseline assessment were included in this cross-network analysis because the eligibility criteria for TechStep required that all participants identified as trans or nonbinary/gender expansive.

#### TechStep Procedures

The TechStep study was conducted at five Subject Recruitment Venues (SRV) located in New York City, Philadelphia, Boston, Houston, and Los Angeles. Participants were recruited via (1) online ads; (2) flyers; (3) street- and venue-based outreach; (4) direct SRV referral or online screener; and (5) participant/peer referral. Following consent, participants completed the baseline ACASI assessment. Immediately following baseline procedures, participants were randomized 1:1:1 into one of three technology-based conditions: (1) WebApp (i.e., a website intervention optimized for a smartphone); (2) text messaging; or (3) an information only website. At month 3, participants who did not show improvement on the primary outcomes were re-randomized to potentially “step-up” to receive virtual e-coaching sessions. The study was approved by the Institutional Review Boards at the University of North Carolina, Chapel Hill, NC, which served as the IRB of Record for all participating institutions and SRVs.

## Measures

### Gender Identity and Sex Assigned at Birth

The independent variable for this analysis was *gender identity*, which was operationalized as a four-category variable: 1 = Trans woman/feminine, 2 = Nonbinary assigned male at birth (AMAB), 3 = Nonbinary assigned female at birth (AFAB), 4 = Trans man/masculine. Response options for gender identity differed between CARES (select one identity term) and TechStep (select all that apply). To ensure that categories were consistent across both studies, we created gender categorization rules for grouping individuals who selected more than one gender identity (See Table 1), accounting for both the participant’s current *gender identity* and *sex assigned at birth*. 72% of participants in the pooled sample selected one gender identity and 28% selected two or more identities.

**Table 1:** Gender categorization for ATN cross-network analysis of CARES and TechStep, 2017–2021, (N = 477)

Assigned sex at birth	Current gender identity	Coding
Male	Male, Man, OR Masculine	Cis Man
Female	Female, Woman, OR Feminine	Cis Woman
Male	Female, Woman, OR Feminine	Trans Woman/Feminine
Male	Trans Female, Trans Woman, OR Trans Feminine	Trans Woman/Feminine
Male	Trans Female AND Female, no other identity	Trans Woman/Feminine
Female	Male, Man, OR Masculine	Trans Man/Masculine
Female	Trans Male AND Male, no other identity	Trans Man/Masculine
Female	Trans Male, Trans Man, OR Trans Masculine	Trans Man/Masculine
Male	Male AND any other identity	Nonbinary AMAB
Male	(Female OR Trans Female) AND any other identity	Nonbinary AMAB
Male	Nonbinary +	Nonbinary AMAB
Female	Female AND any other identity	Nonbinary AFAB
Female	(Male OR Trans Male) AND any other identity	Nonbinary AFAB
Female	Nonbinary +	Nonbinary AFAB

*ATN* Adolescent Medicine Trials Network for HIV/AIDS Interventions, *AMAB* Assigned Male at Birth, *AFAB* Assigned Female at Birth

### PrEP Uptake

The outcome for this analysis was *PrEP uptake*, which was operationalized as a dichotomous variable indicating if the participant had ever taken PrEP in their lifetime (Yes = 1, No = 0). Participants were also asked about current PrEP use (Yes = 1, No = 0), but this variable could not be used as an outcome for the analysis due to lack of variation, as only 7% of respondents reported currently taking PrEP at the time of baseline interview. Current PrEP use was included in the descriptive sample characteristics.

### Mediators

**Fig. 1:**
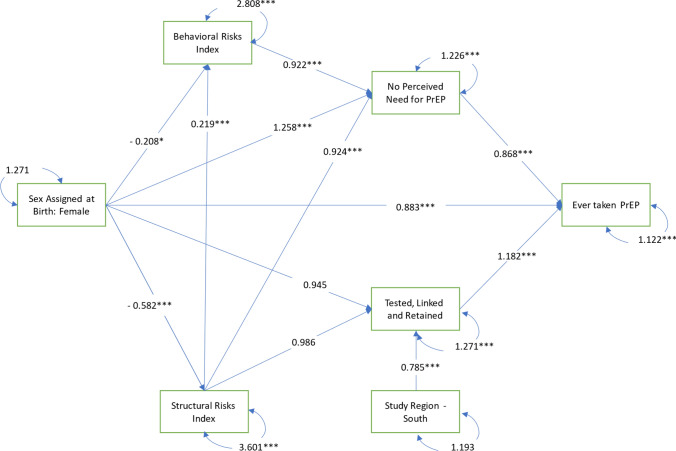
Generalized structural equation model for PrEP uptake among TGEYYA, model 1: unstratified, ATN cross-network analysis of CARES and TechStep, 2017–2021 (N = 477)

**Fig. 2:**
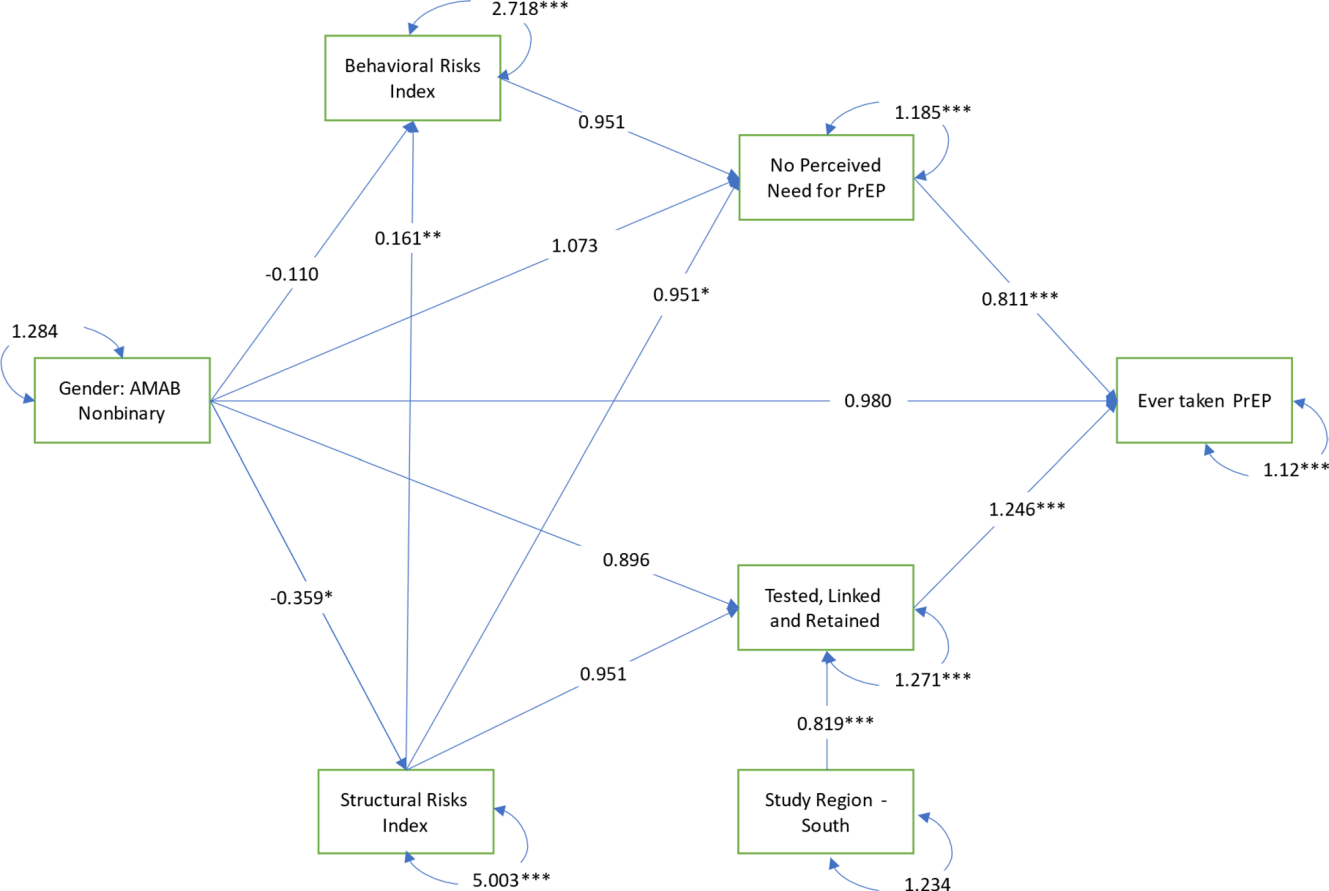
Generalized structural equation model for PrEP uptake among TGEYYA, model 2: stratified, assigned male at birth only, ATN cross-network analysis of CARES and TechStep, 2017-2021, (n = 191)

**Fig. 3:**
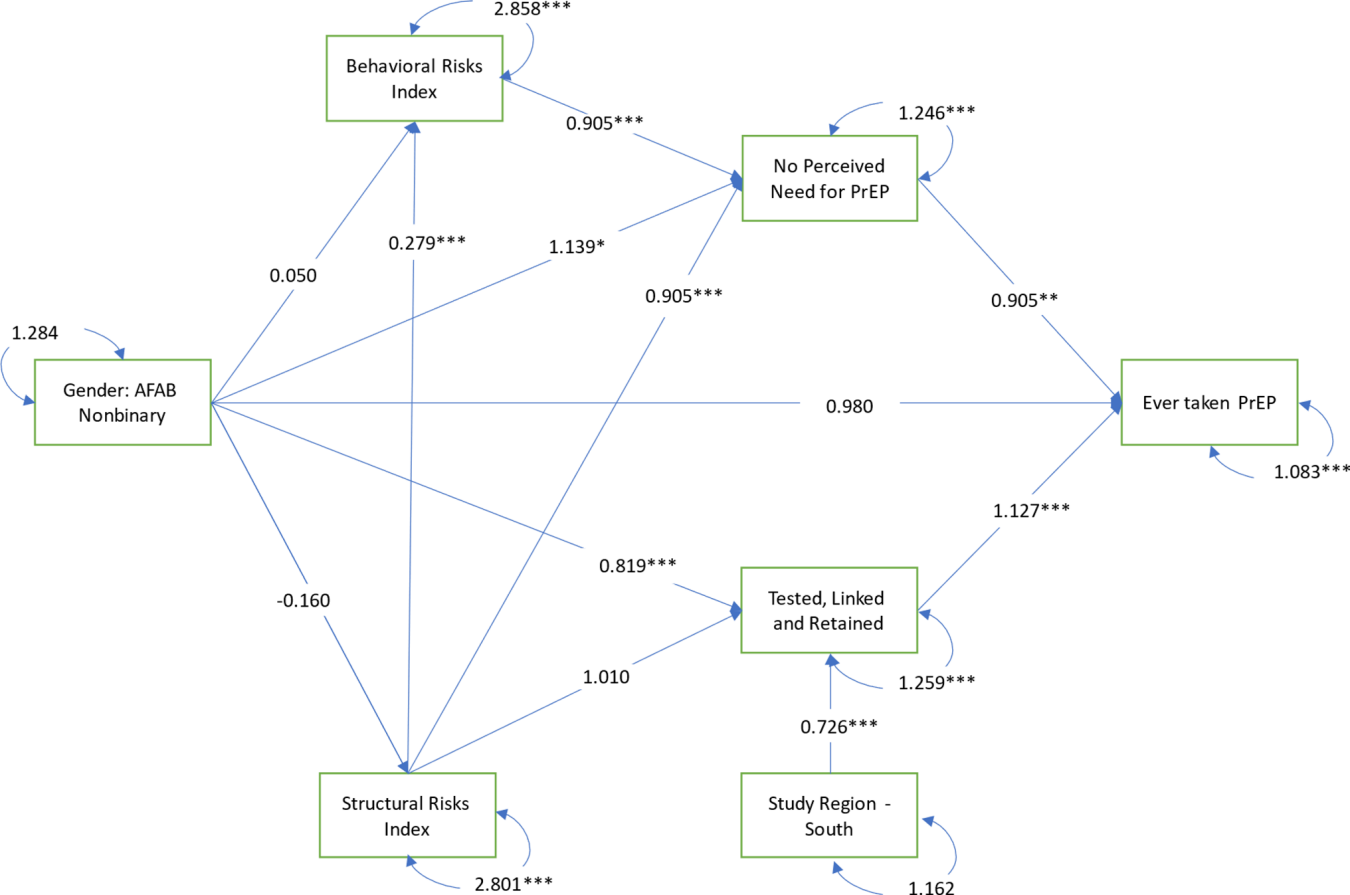
Generalized structural equation model for PrEP uptake among TGEYYA, model 3: stratified, assigned female at birth only, ATN cross-network analysis of CARES and TechStep, 2017-2021, (n = 286)

Four mediators were tested in this analysis (*structural risks*, *behavioral risks*, *no perceived need for PrEP*, and *healthcare utilization*). These mediators were arranged serially along two pathways between gender identity and PrEP uptake to determine if PrEP disparities were explained by differences in both individual risk behaviors and perceptions, as well as by contextual environments and healthcare utilization. These two mediation pathways were designed based on Link and Phelan’s (1995) Theory of Social Conditions as Fundamental Causes [75], such that upstream structural risks were hypothesized to influence both the individual, behavioral/perception pathway and the contextual/healthcare utilization pathway. On the first pathway, we tested whether *structural risks* lead to *behavioral risks*, which in turn lead to *perceived need for PrEP,* and ultimately to *PrEP uptake.* This individual, behavioral/ perception pathway was informed by value expectancy theory from the Health Belief Model [76, 77]. On the second pathway, we tested whether *structural risks* lead to *healthcare utilization* and ultimately to *PrEP uptake.* This structural pathway was informed by Andersen's Model for Health Services Utilization [78]. See Figs. 1–3 for all mediation pathways tested in this analysis. The operationalization of each mediator is described below.

*Behavioral risk index* was a count variable ranging from 0 to 5 for the number of behavioral risks reported by the participant in the last 3 months among the following dichotomous variables (1 = Yes, 0 = No): 1) condomless anal or vaginal sex, 2) multiple sex partners, 3) exchange sex, 4) intimate partner violence, and 5) polysubstance use.

*No perceived need for PrEP* was a dichotomous risk perception variable drawn from a list of reasons for not taking PrEP or stopping PrEP use. Participants were asked if they agreed with the following statement: “I didn’t think I needed PrEP” (1 = Yes, 0 = No).

*Structural risk index* was a count variable ranging from 0 to 5 for the number of structural vulnerabilities ever experienced by the participant among the following dichotomous variables (1 = Yes, 0 = No): 1) housing instability, 2) incarceration, 3) low income (< $500/month), 4) unemployment (not counting students), and 5) uninsurance. Type of insurance and access to insurance through parents could not be evaluated because insurance questions were not asked consistently across the two studies.

*Healthcare utilization* was a four-category, ordered variable ranging from 0 to 3, constructed from multiple questions related to the participant’s HIV testing history, linkage to health care, and retention in care*. Tested* was operationalized as ever having been tested for HIV in lifetime (1 = Yes, 0 = No). *Linked* was operationalized as currently having a primary care provider or regular source of care (1 = Yes, 0 = No). *Retained* was operationalized as having seen a doctor within the last 6 months (1 = Yes, 0 = No). When combining these variables, the four levels were: 0 = Never tested for HIV; 1 = Tested for HIV, but not linked to care; 2 = Tested for HIV, linked to care, but not retained; 3 = Tested, linked, and retained. The distribution across these four categories was reported in the descriptive sample characteristics. For the mediation analysis, this variable was dichotomized to indicate if the participant was tested, linked, and retained (category 3 vs. categories 0–2) because PrEP users must be confirmed seronegative (tested), have a PrEP prescriber (linked), and be regularly engaged in care for ongoing testing and monitoring (retained). Half of participants in the sample were categorized into this final level (tested, linked, and retained in care).

### Control Variables

Several variables were included to control for confounding of gender identity with PrEP uptake and mediating variables. Demographic characteristics included *sexual identity* (Heterosexual (i.e., straight), Homosexual (i.e., gay or lesbian), Bisexual, or Pansexual/Other), *race/ethnicity* (African American, Latinx, White, or Other), *age* in years ranging from 12 to 24, *city* (Boston, Houston, Los Angeles, New Orleans, New York, or Philadelphia) and *region* (Northeast, West, or South), and *education* (Any College vs. None). *Study* enrollment was also included (CARES or TechStep). Finally, a *PrEP Obstacles Index* was constructed as a count variable ranging from 0 to 5 for the sum of the following dichotomous variables (1 = Yes, 0 = No): 1) never heard of PrEP, 2) concerns regarding PrEP side effects, 3) difficulty with schedule for taking PrEP, 4) difficulty getting PrEP medication, and 5) no one to support PrEP use. Due to lack of variation for each item in the *PrEP Obstacles Index*, these variables could not be included as individual covariates in the multivariate analyses. We also could not assess parental insurance nor parental support for PrEP because these variables were not consistent in both studies.

### Statistical Analysis

Data were combined and analyzed using R statistical software (version 4.2.2; R Foundation for Statistical Computing, Vienna, Austria) [79].

### Descriptive Statistics

We computed univariate statistics on the total pooled sample (N = 477). We calculated frequencies and percentages for categorical variables and means and standard deviations for continuous variables.

### Bivariate Analyses

We conducted separate analyses to test for relationships between each covariate and the outcome variable (*PrEP uptake*), the proximal mediators (*no perceived need for PrEP* and *healthcare utilization*), and the distal mediators (*structural and behavioral risk indices)*. For each categorical control variable, we performed a Pearson's chi-squared test (or Fisher’s exact test for small cell sizes < 5) to determine significant differences in PrEP uptake, no perceived need for PrEP, healthcare utilization, and the structural and behavioral risk indices. For continuous control variables, we performed two-tailed t-tests. The significance level was set at p < 0.05.

### Structural Equation Models

After determining which variables were significantly associated with PrEP uptake and the mediators in bivariate comparisons, we built a generalized structural equation model (GSEM) using the *lavaan* package in R (version 0.6–10) to allow for discrete measures and test all mediation pathways simultaneously. We selected seven variables for the full model, each of which was associated with one or more outcomes/mediators below the significance level of p < 0.05. These variables were: gender identity, region, behavioral risk index, structural risk index, no perceived need for PrEP, healthcare utilization, and PrEP uptake. To ensure convergence, we limited the number of model parameters by dropping control variables which were not associated with PrEP uptake when adjusting for other factors (race/ethnicity, age, and education) and dichotomizing gender identity by sex assigned at birth (1 = Female, 0 = Male) and region (1 = South, 0 = West or Northeast).

We tested three GSEMs: Model 1 with the full pooled sample (N = 477), Model 2 with only participants assigned male at birth (n = 191), and Model 3 with only participants assigned female at birth (n = 286). In the stratified GSEMs, (Models 2 and 3), the independent variable was dichotomized to compare by gender identity among those with the same sex assigned at birth (1 = Nonbinary, 0 = Trans Woman or Trans Man). We reported the exponentiated estimates as adjusted odds ratios (AOR), 95% confidence intervals (CI), and p-values along each path. We calculated the likelihood ratio chi-squared test, goodness of fit index (GFI), comparative fit index (CFI), and root mean squared error of approximation (RMSEA) for each GSEM. Model fit was determined by a chi-squared p-value > 0.05, GFI > 0.95, CFI > 0.90, and RMSEA < 0.05.

As sensitivity analyses, we conducted five separate regressions of PrEP uptake and the four mediators on control variables to determine if coefficient values and statistical significance levels matched equivalent pathways in the GSEM. Because the GSEM and regression results were consistent, only the GSEM results are reported to limit redundancy.

## Results

### Sample Characteristics

See Table 2 for the descriptive characteristics of the sample in total and stratified by PrEP uptake, no perceived need for PrEP, and healthcare utilization.

**Table 2:** Sample characteristics of TGEYYA in total and by PrEP uptake, no perceived need for PrEP, and healthcare utilization, ATN cross-network analysis of CARES and TechStep, 2017–2021, (N = 477)

	Total		PrEP Uptake: Ever Taken PrEP			Risk Perception: No Perceived Need For PrEP			Healthcare Utilization: Tested, Linked, & Retained		
Variable	n / Mean	% / SD	n / Mean	% / SD	P	n / Mean	% / SD	P	n / Mean	% / SD	P
Gender Identity					< 0.001			< 0.000			0.010
Trans Woman/Feminine	96	20.13%	28	29.17%		19	19.79%		55	57.29%	
Nonbinary Assigned Male at Birth (AMAB)	95	19.92%	22	23.16%		28	29.47%		46	48.42%	
Nonbinary Assigned Female at Birth (AFAB)	145	30.40%	9	6.21%		90	62.07%		62	42.76%	
Trans Man/Masculine	141	29.56%	17	12.06%		67	47.52%		84	59.57%	
Sex Assigned At Birth					< 0.001			< 0.001			0.014
Female	286	59.96%	26	9.09%		193	48.13%		146	51.05%	
Male	191	40.04%	50	26.18%		11	14.47%		101	52.88%	
Sexual Identity					< 0.001			0.012			0.625
Heterosexual (Straight)	44	9.22%	15	34.09%		11	25.00%		25	56.82%	
Homosexual (Gay or Lesbian)	125	26.21%	26	20.80%		50	40.00%		66	52.80%	
Bisexual	107	22.43%	15	14.02%		57	53.27%		52	48.60%	
Pansexual or Other	201	42.14%	20	9.95%		86	42.79%		104	51.74%	
Race/Ethnicity					0.007			< 0.001			0.001
African American	93	19.50%	16	17.20%		25	26.88%		47	50.54%	
Latinx	93	19.50%	25	26.88%		25	26.88%		55	59.14%	
White	208	43.61%	28	13.46%		108	51.92%		106	50.96%	
Other	83	17.40%	7	8.43%		46	55.42%		39	46.99%	
Age (Mean, SD)	21.37	1.97	21.37	1.94	0.388	21.5	2.02	0.241	21.54	1.92	0.018
15–17 years old 18–19 years old 20–21 years old 22–24 years old	41 91 181 164	8.60% 19.08% 37.95% 34.38%	6 20 26 24	14.63% 21.98% 14.36% 14.63%		15 35 73 81	36.59% 38.46% 40.33% 49.39%		17 49 90 91	41.46% 53.85% 49.72% 55.49%	
Education					0.189			0.009			0.237
Any College Or Above	354	74.21%	53	14.97%		167	47.18%		187	52.82%	
High School Diploma/GED	98	20.55%	17	17.35%		32	32.65%		48	48.98%	
Less Than High School	25	5.24%	6	24.00%		5	20.00%		12	48.00%	
Study Region					0.133			< 0.001			< 0.001
Northeast	174	36.48%	25	14.37%	0.156	104	59.77%	< 0.001	94	54.02%	< 0.001
Boston	59	33.91%	9	15.25%		32	54.24%		35	59.32%	
New York City	67	38.51%	13	19.40%		44	65.67%		42	62.69%	
Philadelphia	48	27.59%	3	6.25%		28	58.33%		17	35.42%	
South	109	22.85%	13	11.93%	0.065	45	41.28%	< 0.001	37	33.94%	< 0.001
Houston	57	52.29%	4	7.02%		28	49.12%		15	26.32%	
New Orleans	52	47.71%	9	17.31%		17	32.69%		22	42.31%	
West	194	40.67%	38	19.59%		55	28.35%		116	59.79%	
Los Angeles	194	100.00%	38	19.59%		55	28.35%		116	59.79%	
Study Enrollment					0.209			< 0.001			0.009
CARES	197	41.30%	37	18.78%		47	23.86%		110	55.84%	
TechStep	280	58.70%	39	13.93%		157	56.07%		137	48.93%	
Ever Taken PrEP								< 0.001			< 0.001
No	401	84.07%				193	48.13%		187	46.63%	
Yes	76	15.93%				11	14.47%		60	78.95%	
Current PrEP Use					< 0.001			< 0.001			< 0.001
No	442	92.66%	43	9.73%		203	45.93%		216	48.87%	
Yes	35	7.34%	33	100.00%		0	0.00%		31	88.57%	
No Perceived Need For PrEP					< 0.001						0.027
No	273	57.23%	65	23.81%					145	53.11%	
Yes	204	42.77%	11	5.39%					102	50.00%	
Healthcare Utilization					< 0.001			0.027			
Never Tested For HIV	96	20.13%	1	1.04%		52	54.17%				
Tested, Not Linked To Care	87	18.24%	6	6.90%		29	33.33%				
Tested, Linked To Care, Not Retained	47	9.85%	9	19.15%		21	44.68%				
Tested, Linked To Care, And Retained	247	51.78%	60	24.29%		102	41.30%				
Behavioral Risk Index (Mean, SD)	1.56	1.06	1.89	0.97	0.003	1.25	0.89	< 0.001	1.63	1.03	< 0.001
0	70	14.68%	6	8.57%		40	57.14%		32	45.71%	
1	172	36.06%	17	9.88%		93	54.07%		84	48.84%	
2	158	33.12%	38	24.05%		53	33.54%		88	55.70%	
3 +	52	10.90%	15	19.48%		18	23.38%		43	55.84%	
Exchange Sex, Last 3 Months					0.508			< 0.001			0.006
No	422	88.47%	65	15.40%		197	46.68%		217	51.42%	
Yes	55	11.53%	11	20.00%		7	12.73%		30	54.55%	
Multiple Sex Partners, Last 3 Months					0.002			< 0.001			< 0.001
No	260	54.51%	29	11.15%		140	53.85%		123	47.31%	
Yes	217	45.49%	47	21.66%		64	29.49%		124	57.14%	
Condomless Sex, Last 3 Months					1.000			0.978			0.365
No	206	43.19%	33	16.02%		87	42.23%		113	54.85%	
Yes	271	56.81%	43	15.87%		117	43.17%		134	49.45%	
Poly Substance Use, Last 3 Months					0.144			0.019			< 0.001
No	312	65.41%	44	14.10%		146	46.79%		155	49.68%	
Yes	165	34.59%	32	19.39%		58	35.15%		92	55.76%	
Violence, Last 3 Months					0.163			0.012			0.186
No	437	91.61%	66	15.10%		195	44.62%		225	51.49%	
Yes	40	8.39%	10	25.00%		9	22.50%		22	55.00%	
Structural Risk Index (Mean, SD)	1.26	1.26	1.43	1.24	0.332	0.99	0.93	< 0.001	1.14	1.13	< 0.001
0	129	27.04%	28	16.28%		97	56.40%		89	51.74%	
1	163	34.17%	25	16.45%		68	44.74%		81	53.29%	
2	90	18.87%	8	9.41%		30	35.29%		41	48.24%	
3 +	64	13.42%	16	22.22%		10	13.89%		38	52.78%	
Housing Instability					0.233			< 0.001			0.001
No	386	80.92%	58	15.03%		192	49.74%		193	50.00%	
Yes	91	19.08%	18	19.78%		12	13.19%		54	59.34%	
Incarceration					1.000			< 0.001			0.272
No	428	89.73%	68	15.89%		196	45.79%		223	52.10%	
Yes	49	10.27%	8	16.33%		8	16.33%		24	48.98%	
Employment					0.356			0.002			0.088
Employed	156	32.70%	30	19.23%		58	37.18%		89	57.05%	
Student	178	37.32%	24	13.48%		94	52.81%		90	50.56%	
Unemployed/Disabled	143	29.98%	22	15.38%		52	36.36%		68	47.55%	
Income					1.000			0.002			0.199
< $500	209	43.82%	34	16.27%		71	33.97%		108	51.67%	
> = $500	268	56.18%	42	15.67%		133	49.63%		139	51.87%	
Insurance					1.000			0.001			0.001
No	61	12.79%	10	16.39%		14	22.95%		28	45.90%	
Yes	416	87.21%	66	15.87%		190	45.67%		219	52.64%	
PrEP Obstacles Index (Mean, SD)	1.71	0.98	1.8	1.02	< 0.001	2.45	0.68	< 0.001	1.69	0.89	< 0.001
0	48	10.06%	0	0.00%		0	0.00%		12	25.00%	
1	149	31.24%	58	38.93%		0	0.00%		100	67.11%	
2	193	40.46%	16	8.29%		129	66.84%		95	49.22%	
3 +	70	14.68%	2	2.86%		75	86.21%		40	45.98%	
Difficulty With Schedule For Taking PrEP					0.071			1.000			0.116
No	445	93.29%	75	16.85%		190	42.70%		237	53.26%	
Yes	32	6.71%	1	3.13%		14	43.75%		10	31.25%	
Concerns Regarding PrEP Side Effects					0.008			0.008			0.608
No	369	77.36%	68	18.43%		145	39.30%		196	53.12%	
Yes	108	22.64%	8	7.41%		59	54.63%		51	47.22%	
Difficulty Getting Medication					0.034			0.864			0.327
No	449	94.13%	76	16.93%		193	42.98%		236	52.56%	
Yes	28	5.87%	0	0.00%		11	39.29%		11	39.29%	
No One To Support Your PrEP Use					0.153			0.168			0.734
No	461	96.65%	76	16.49%		194	42.08%		237	51.41%	
Yes	16	3.35%	0	0.00%		10	62.50%		10	62.50%	
Ever Heard Of PrEP					0.003			< 0.001			< 0.001
No	48	10.06%	0	0.00%		0	0.00%		12	25.00%	
Yes	429	89.94%	76	17.72%		204	47.55%		235	54.78%	

*TGEYYA* Transgender and Gender Expansive Youth and Young Adults, *PrEP* Pre-exposure Prophylaxis, *ATN* Adolescent Medicine Trials Network for HIV/AIDS Interventions, *AMAB* Assigned Male at Birth, *AFAB* Assigned Female at Birth

### Demographics

60% of the sample were assigned female at birth and 40% were assigned male at birth. Within each group, half identified as trans (trans woman or trans man), and half identified as nonbinary. Most participants (65%) identified as pansexual or bisexual, 26% identified as homosexual (i.e., gay or lesbian), and 9% identified as heterosexual (i.e., straight). For race/ethnicity, 44% were White, 20% African American/Black, 20% Latinx, and 17% Other. The mean age was 21 years old (SD: 2.0, range: 15–24), and the majority of participants were over 18 years of age (91%). Most had at least some college education (74%). By region, 37% of participants were recruited in the Northeast from Boston, New York City, or Philadelphia; 23% in the South from Houston or New Orleans; and 41% in the West from Los Angeles. Slightly more than half of participants (59%) were enrolled in the TechStep study and 41% in CARES.

### PrEP Uptake

Only 16% percent of participants reported ever taking PrEP in their lifetime, and 7% reported that they were currently taking PrEP at study enrollment. In bivariate comparisons, the proportion who reported PrEP uptake in their lifetime was significantly higher among: trans women (29%) and nonbinary AMAB participants (23%) compared to trans men (12%) and nonbinary AFAB participants (6%); heterosexual and homosexual participants compared to bisexual and pansexual participants; Latinx participants; those who reported a perceived need for PrEP; those who had been tested, linked, and retained in health care; those who reported two or more behavioral risks, and in particular, multiple sex partners; those who did not report concerns regarding PrEP side effects or difficulty getting the medication; and those who had heard of PrEP.

### No Perceived Need for PrEP

Almost half of participants (43%) reported no perceived need for PrEP. The proportion of participants reporting no perceived need for PrEP was significantly higher among: nonbinary AFAB participants and trans men vs. nonbinary AMAB participants and trans women; bisexual, pansexual, and homosexual participants compared to heterosexual participants; White participants and those of other races/ethnicities compared to African American/Black and Latinx participants; those with any college education; those recruited in the Northeast; those enrolled in TechStep vs. CARES; those who had never taken PrEP; those who had never been tested for HIV; those who reported fewer behavioral risks and fewer structural risks; and those who reported two or more PrEP obstacles, especially concerns regarding PrEP side effects.

### Healthcare Utilization

Half of participants (52%) reported that they were tested, linked and retained in healthcare. Among those who were not retained in care, 20% had never been tested for HIV, 18% had been tested but did not have a regular source of medical care, and 10% had been tested and linked to medical care but had not seen a doctor in the last 6 months. The proportion of participants who were tested, linked, and retained in care was significantly higher among: trans women and trans men vs. nonbinary participants, irrespective of sex assigned at birth; Latinx participants; older participants; those recruited in the Northeast or West compared to the South; those enrolled in CARES vs. TechStep; those who had ever taken PrEP; those who reported perceived need for PrEP; those who reported two or more behavioral risks, especially exchange sex, multiple sex partners, and polysubstance use; those who had insurance; and those who had heard of PrEP.

### Behavioral Risk Index

Participants reported an average of 1.6 behavioral risks (SD: 1.1) in the last 3 months, and 44% reported two or more behavioral risks. The majority of participants who reported two or more behavioral risks were assigned male at birth. The most commonly reported behavioral risks were condomless anal or vaginal sex (57%), having multiple sex partners (45%), and polysubstance use (35%). Exchange sex and violence were each reported by fewer than 12% of participants, but more commonly reported by trans women (23% and 16%, respectively). 67% percent of nonbinary participants assigned male at birth, 58% of trans women, 56% of trans men, and 49% of nonbinary participants assigned female at birth reported condomless anal or vaginal sex. There were no significant differences in those reporting multiple sex partners across all gender identities. Trans women reported significantly more behavioral risks on average compared to nonbinary participants assigned male at birth, trans men, and nonbinary participants assigned female at birth.

### Structural Risk Index

Participants reported an average of 1.3 structural risks (SD: 1.3) in their lifetime, and 32% reported experiencing two or more structural risks. Most participants who reported two or more structural risks were assigned male at birth. The most commonly reported structural risks were being low income (44%), unemployed or disabled (30%), and housing instability (19%). Incarceration and being uninsured were each reported by fewer than 13% of participants. Trans women reported significantly more structural risks on average compared to trans men and nonbinary participants assigned female at birth.

### PrEP Obstacles Index

Participants reported an average of 1.7 PrEP obstacles (SD: 1.0), and just over half (55%) reported two or more obstacles that contributed to them not using PrEP. The most commonly reported obstacle was concerns regarding PrEP side effects (23%). No other PrEP obstacle was reported by more than 10% of respondents. The vast majority of participants had heard of PrEP before the study (90%).

### Generalized Structural Equation Models

The results of the GSEM analyses are displayed in Tables 3, 4, 5 and visualized in the diagrams in Figs. 1–3. As hypothesized, sex assigned at birth was directly associated with PrEP uptake and directly associated with all mediators in the model except healthcare utilization. Furthermore, all paths in Model 1 with the full pooled sample (see Fig. 1) were significant except the paths from sex assigned at birth and the structural risk index to healthcare utilization. Compared to participants assigned male at birth, participants assigned female at birth reported significantly fewer behavioral risks (Coef: −0.208, P Value: 0.032, 95% CI: −0.400 – −0.020), fewer structural risks (Coef: −0.582 P Value < 0.001, 95% CI: −0.800 – −0.360), were more likely to report no perceived need for PrEP (AOR: 1.258, P Value < 0.001, 95% CI: 1.150 – 1.363), and were less likely to have ever taken PrEP (AOR: 0.883, P Value < 0.001, 95% CI: 0.827 – 0.942).

**Table 3:** Generalized structural equation model of PrEP uptake among TGEYYA, unstratified, ATN cross-network analysis of CARES and TechStep, 2017-2021, model 1: full sample, (N = 477)

From	To	Estimate	P	95% CI Left	95% CI Right
Sex Assigned At Birth: Female	Structural Risk Index	−0.582***	< 0.001	−0.800	−0.360
Structural Risk Index	Behavioral Risk Index	0.219***	< 0.001	0.140	0.300
Sex Assigned At Birth: Female	Behavioral Risk Index	−0.208*	0.032	−0.400	−0.020
Structural Risk Index	Healthcare Utilization	0.986	0.484	0.951	1.030
Region: South	Healthcare Utilization	0.785***	< 0.001	0.712	0.869
Sex Assigned At Birth: Female	Healthcare Utilization	0.945	0.229	0.861	1.041
Behavioral Risk Index	No Perceived Need For PrEP	0.922***	< 0.001	0.887	0.961
Structural Risk Index	No Perceived Need For PrEP	0.924***	< 0.001	0.896	0.951
Sex Assigned At Birth: Female	No Perceived Need For PrEP	1.258***	< 0.001	1.150	1.363
No Perceived Need For PrEP	Ever Taken PrEP	0.868***	< 0.001	0.819	0.923
Healthcare Utilization	Ever Taken PrEP	1.182***	< 0.001	1.116	1.259
Sex Assigned At Birth: Female	Ever Taken PrEP	0.883***	< 0.001	0.827	0.942
Model Fit Statistics:					
Chi Squared P-value	0.014				
GFI	0.990				
CFI	0.982				
RMSEA	0.033				
AGFI	0.965				
SRMR	0.031				
RFI	0.882				
NFI	0.953				
NNFI	0.956				
IFI	0.983				
AIC	4464.483				
BIC	4535.331				

*PrEP* Pre-Exposure Prophylaxis, *TGEYYA* Transgender and Gender Expansive Youth and Young Adults, *ATN* Adolescent Medicine Trials Network for HIV/AIDS Interventions, *AMAB* Assigned Male at Birth, *AFAB* Assigned Female at Birth *CI* Confidence Interval, *GFI/AGFI* (Adjusted) Goodness of Fit, *CFI* Comparative Fit Index, *RMSEA*: Root Mean Square Error of Approximation, *SRMR*: Standardized Root Mean Square Residual, *RFI*: Relative Fit Index, *NFI/NNFI* (Non) Normed Fit index, *IFI* Incremental Fit Index, *AIC* Akaike Information Criterion, *BIC* The Bayesian Information Criterion

Significance Levels: *p < 0.05, **p < 0.01, ***p < 0.001

**Table 4:** Generalized structural equation model for PrEP uptake among TGEYYA, stratified by sex assigned at birth, ATN cross-network analysis of CARES and TechStep, 2017-2021, model 2: assigned male at birth only, (n = 191)

From	To	Estimate	P	95% CI Left	CI Right
Gender: Nonbinary AMAB	Structural Risk Index	− 0.359*	0.048	-0.720	0.000
Structural Risk Index	Behavioral Risk Index	0.161**	0.003	0.060	0.260
Gender: Nonbinary AMAB	Behavioral Risk Index	− 0.110	0.433	-0.390	0.170
Structural Risk Index	Healthcare Utilization	0.951	0.055	0.905	1.000
Region: South	Healthcare Utilization	0.819**	0.007	0.705	0.951
Gender: Nonbinary AMAB	Healthcare Utilization	0.896	0.130	0.787	1.030
Behavioral Risk Index	No Perceived Need For PrEP	0.951	0.058	0.905	1.000
Structural Risk Index	No Perceived Need For PrEP	0.951*	0.015	0.905	0.990
Gender: Nonbinary AMAB	No Perceived Need For PrEP	1.073	0.275	0.951	1.209
No Perceived Need For PrEP	Ever Taken PrEP	0.811***	< 0.001	0.726	0.914
Healthcare Utilization	Ever Taken PrEP	1.246***	< 0.001	1.116	1.405
Gender: Nonbinary AMAB	Ever Taken PrEP	0.980	0.735	0.869	1.105
Model Fit Statistics					
Chi Squared P-value	0.304				
GFI	0.981				
CFI	0.970				
RMSEA	0.031				
AGFI	0.933				
SRMR	0.046				
RFI	0.657				
NFI	0.863				
NNFI	0.925				
IFI	0.976				
AIC	1886.867				
BIC	1942.155				

*PrEP* Pre-Exposure Prophylaxis, *TGEYYA* Transgender and Gender Expansive Youth and Young Adults, *ATN* Adolescent Medicine Trials Network for HIV/AIDS Interventions, *AMAB* Assigned Male at Birth, *AFAB* Assigned Female at Birth, *CI* Confidence Interval, *GFI/AGFI* (Adjusted) Goodness of Fit, *CFI* Comparative Fit Index, *RMSEA* Root Mean Square Error of Approximation, *SRMR* Standardized Root Mean Square Residual, *RFI* Relative Fit Index, *NFI/NNFI* (Non) Normed Fit Index, *IFI* Incremental Fit Index, *AIC* Akaike Information Criterion, *BIC* The Bayesian Information Criterion

Significance Levels: *p < 0.05, **p < 0.01, ***p < 0.001

**Table 5:** Generalized structural equation model for PrEP uptake among TGEYYA, stratified by sex assigned at birth, ATN cross-network analysis of CARES and TechStep, 2017-2021, model 3: assigned female at birth only, (n = 286)

From	To	Estimate	P	95% CI Left	95% CI Right
Gender: Nonbinary AFAB	Structural Risk Index	−0.160	0.187	−0.390	0.080
Structural Risk Index	Behavioral Risk Index	0.279***	< 0.001	0.160	0.400
Gender: Nonbinary AFAB	Behavioral Risk Index	0.050	0.706	−0.190	0.280
Structural Risk Index	Healthcare Utilization	1.010	0.661	0.961	1.073
Region: South	Healthcare Utilization	0.726***	< 0.001	0.631	0.835
Gender: Nonbinary AFAB	Healthcare Utilization	0.819***	0.001	0.733	0.914
Behavioral Risk Index	No Perceived Need For PrEP	0.905***	< 0.001	0.861	0.951
Structural Risk Index	No Perceived Need For PrEP	0.905***	< 0.001	0.861	0.951
Gender: Nonbinary AFAB	No Perceived Need For PrEP	1.139*	0.018	1.020	1.271
No Perceived Need For PrEP	Ever Taken PrEP	0.905**	0.003	0.844	0.970
Healthcare Utilization	Ever Taken PrEP	1.127***	< 0.001	1.051	1.197
Gender: Nonbinary AFAB	Ever Taken PrEP	0.980	0.479	0.914	1.041
Model Fit Statistics					
Chi Squared P-value	0.497				
GFI	0.990				
CFI	1.000				
RMSEA	0.001				
AGFI	0.964				
SRMR	0.030				
RFI	0.845				
NFI	0.938				
NNFI	1.016				
IFI	1.006				
AIC	2516.714				
BIC	2578.866				

*PrEP* Pre-Exposure Prophylaxis, *TGEYYA* Transgender and Gender Expansive Youth and Young Adults, *ATN* Adolescent Medicine Trials Network for HIV/AIDS Interventions, *AMAB* Assigned Male at Birth, *AFAB* Assigned Female at Birth, *CI* Confidence Interval, *GFI/AGFI* (Adjusted) Goodness of Fit, *CFI* Comparative Fit Index, *RMSEA* Root Mean Square Error of Approximation, *SRMR* Standardized Root Mean Square Residual, *RFI* Relative Fit Index, *NFI/NNFI* (Non) Normed Fit Index, *IFI* Incremental Fit Index, *AIC* Akaike Information Criterion, *BIC* The Bayesian Information Criterion

Significance Levels: *p < 0.05, **p < 0.01, ***p < 0.001

In addition to the direct effects observed between sex assigned at birth and PrEP uptake, structural and behavioral risks significantly mediated the relationship between sex assigned at birth and perceived need for PrEP, which, in turn mediated the relationship between structural and behavioral risks and PrEP uptake. Those reporting more structural risks reported more behavioral risks (Coef: 0.219, P Value < 0.001, 95% CI: 0.140–0.300) and were less likely to report no perceived need for PrEP (AOR: 0.924, P Value < 0.001, 95% CI: 0.896 – 0951). Those who reported more behavioral risks were also less likely to report no perceived need for PrEP (AOR: 0.992, P Value < 0.001, 95% CI: 0.887–0.961), and those who reported no perceived need for PrEP were less likely to have ever taken PrEP (OR: 0.868, P Value < 0.001, 95% CI: 0.819–0.923).

Healthcare utilization significantly mediated the relationship between region and PrEP uptake but not between sex assigned at birth and PrEP uptake. Those who were tested, linked, and retained in care were more likely to have ever taken PrEP (AOR: 1.182, P Value < 0.001, 95% CI: 1.116–1.259), and those in the South were less likely to be tested, linked, and retained in care (AOR: 0.785 P Value < 0.001, 95% CI: 0.712–0.869).

When stratifying the model to include only those assigned male at birth (see Fig. 2), there were no significant differences observed between nonbinary participants assigned male at birth and trans women in behavioral risks, perceived need for PrEP, healthcare utilization, nor PrEP uptake. The only direct difference observed between these two groups was that nonbinary participants reported marginally fewer structural risks than trans women (AOR: −0.359, P Value: 0.048, 95% CI: −0.100–0.001).

As observed in Model 1 with the full pooled sample, those reporting more structural risks again reported more behavioral risks (Coef: 0.161, P Value: 0.003, CI: 0.060–0.260) and were less likely to report no perceived need for PrEP (AOR: 0.951, P Value: 0.015, CI: 0.905–0.990); those with no perceived need for PrEP were less likely to have ever taken PrEP (AOR: 0.811, P Value < 0.001, 95% CI: 0.726–0.914); those who were tested, linked, and retained in care were more likely to have ever taken PrEP (AOR: 1.246, P Value < 0.001, 95% CI: 1.116–1.405), and those in the South were less likely to be tested, linked, and retained in care (AOR: 0.819, P Value: 0.007, 95% CI: 0.705–0.951).

When stratifying the model to include only those assigned female at birth (see Fig. 3), there were no significant differences between nonbinary participants and trans men in behavioral and structural risks nor PrEP uptake. However, nonbinary participants were more likely to report no perceived need for PrEP (AOR: 1.139, P Value: 0.018, 95% CI: 1.020–1.271) and less likely to be tested, linked, and retained in care (AOR: 0.819, P Value: 0.001, 95% CI: 0.733–0.914) than trans men.

Again as observed in Model 1 with the full pooled sample, those who reported more structural risks also reported more behavioral risks (Coef: 0.279, P Value < 0.001, 95% CI: 0.160–0.400) and were less likely to report no perceived need for PrEP (AOR: 0.905, P Value < 0.000; 95% CI: 0.861–0.951); those who reported more behavioral risks were less likely to report no perceived need for PrEP (AOR: 0.905, P Value < 0.000, 95% CI: 0.861- 0.951); those who reported no perceived need for PrEP were less likely to have ever taken PrEP (AOR: 0.905, P Value: 0.003, 95% CI: 0.844–0.970); those who were tested, linked, and retained in care were more likely to have ever taken PrEP (AOR: 1.127, P Value < 0.001, 95% CI: 1.051–1.197); and participants from the South were less likely to be tested, linked, and retained in care (AOR: 0.726, P Value < 0.001, C95% I: 0.631–0.835).

## Discussion

Findings from the current study identified potential mechanisms associated with disparities in PrEP uptake among 477 TGEYYA enrolled in two protocols in the National Adolescent Trials Network. Most participants in this sample would be considered PrEP candidates based on CDC guidelines [80, 81], due to recent sexual and substance use behaviors that increase the likelihood of HIV transmission; 85% reported at least one HIV risk behavior in the last 3 months. Most participants reported condomless vaginal or anal sex, almost half reported having multiple sex partners, and a third reported polysubstance use. Almost all participants were aware of PrEP as an HIV prevention option, but less than 1 in 3 participants assigned male at birth and 1 in 6 participants assigned female at birth had ever taken PrEP.

There was significantly higher lifetime PrEP uptake reported among those assigned male at birth compared to those assigned female at birth. Structural and behavioral risks and perceived need for PrEP mediated the relationship between sex assigned at birth and PrEP uptake, as hypothesized along the behavioral/perception pathway. Trans women reported the highest average number of both behavioral and structural risks of all gender identities. For those assigned female at birth, PrEP uptake was higher among trans men than nonbinary participants. Despite reporting lower behavioral and structural risks compared to those assigned male at birth, trans men and nonbinary participants assigned female at birth were engaging in some HIV transmission behaviors – such as having multiple concurrent sex partners – yet their perceived need for PrEP and actual use of PrEP was very low. This might be due in part to higher perceived susceptibility for HIV infection being associated with having partners who are cis male or perceiving one’s sexual or social networks to be high risk [17, 33]; however, this study was limited in its ability to assess peer influences and partner characteristics of participants, such as partner gender identity and sex assigned at birth due to incongruity in these partner variables between the CARES and TechStep studies. Social network and partner characteristics of TGEYYA will be important to investigate in future research on PrEP use among adolescents.

TGEYYA experience heightened susceptibility for HIV infection, yet our estimates of 16% lifetime PrEP uptake and 7% current PrEP use are lower than many other PrEP uptake estimates nationally. In 2021, the CDC estimated that 25% of the 1.2 million eligible people with indication for PrEP use received a PrEP prescription [81]. Our findings on PrEP uptake are also lower than a CDC study estimating 33.5% of trans women aged 18–29 years old had indicated PrEP use [82]. The inclusion of younger adolescents in our sample might contribute to these lower rates of PrEP uptake, as seen in other groups with PrEP indications, in which younger people have lower uptake [83]. Our findings are more in line with preliminary CDC data showing lower PrEP uptake among adolescents compared to adults [84]. Several barriers to PrEP use among adolescents and young adults have been described, including barriers related to cost and insurance, fear of disclosing gender identity or sexual orientation to parents and healthcare providers, and lower risk perceptions [85–87]. Yet, most of what is known about barriers and facilitators for PrEP use among adolescents are from cisgender male and trans women samples [15, 87]. For trans men and nonbinary adolescents and young adults who are PrEP candidates, more research is needed to understand their unique barriers to care and PrEP uptake.

Trans women reported the highest average number of both behavioral and structural risks of all TGEYYA, highlighting the importance of addressing syndemics among trans women to reduce new HIV infections among this highly impacted population. Our findings also demonstrate the need to intervene upstream (e.g., policies that allow gender affirming care in healthcare settings, anti-discrimination laws, etc.) to alter structural conditions that lead to elevated structural vulnerabilities and sexual and substance use behaviors among trans women [41, 42, 88]. This coincides with extensive research examining syndemic correlates of HIV transmission [3, 12, 27, 52], unmet structural needs [89], and PrEP uptake [48, 60, 90, 91] among trans women. Trans women experience many disparities in structural factors (e.g., discrimination, housing, incarceration), which impact behavioral risk (e.g., substance use, multiple sex partners, exchange sex, and condomless anal sex) for HIV transmission [42, 88].

HIV testing and prevention services often seek to engage trans women because they experience inequities in HIV infection rates [92]. Since HIV testing and linkage to care services would associate behavioral risks with the need for PrEP, those engaged in care might be more likely to view themselves as a PrEP candidate to reduce their HIV transmission risk [50]. Our findings confirm that sexual risk behaviors and perceived need for PrEP are important drivers for PrEP uptake generally [93, 94], and for trans women specifically [91]. Similar findings of higher PrEP uptake among trans women compared to trans men and nonbinary individuals have been found in national samples of young gender minorities [95], which is consistent with early PrEP trials, rollout, and marketing campaigns being focused almost exclusively on trans women compared to other gender minority groups [60]. However, these results contradict two previous studies, in which trans masculine youth were more willing to or more likely to be on PrEP compared to trans feminine youth [14, 96].

These results also highlight important information for nonbinary adolescents assigned male at birth. Our results found that nonbinary AMAB participants reported higher occurrence of condomless anal or vaginal sex compared to trans women and no difference in reporting multiple partners or polysubstance use. Yet trans women reported significantly more structural risks, which may be contributing to slightly higher mean number of behavioral risks among trans women compared to nonbinary AMAB participants. Emerging research comparing binary and nonbinary trans youth have found similar behavioral risk profiles between these gender identity groups[6]. Yet, few studies specifically look at the unique needs of nonbinary youth assigned male at birth and instead combine them with nonbinary youth assigned female at birth [89].

These findings also contribute to emerging research on PrEP uptake among adolescents assigned female at birth. While trans men reported fewer numbers of behavioral risks than trans women, a majority reported condomless vaginal or anal sex and almost half had multiple sex partners in the last 3 months. There were no significant differences across the four gender identity groups for having multiple sex partners. In comparison to trans women, trans men and nonbinary individuals assigned female at birth are understudied in research examining HIV transmission risk and PrEP uptake [48]. Researchers have often erroneously assumed that most trans men practice sexual behaviors that do not put them at risk for HIV due to conflation of gender identity and sexual identity assuming heteronormative preferences (e.g., trans men assumed to have a sexual preference for cisgender women) [58, 97]. However, for trans men who have sex with cisgender men or trans women and practice condomless sex in the absence of PrEP use, risk for HIV is elevated to the same extent as cisgender men who have sex with men [54, 98]. Recent research suggests that trans men also engage in elevated substance use behaviors and exhibit syndemic mental health conditions, including high levels of depression and anxiety, all of which are associated with increased sexual risk behaviors and high STI incidence [55, 56]. Despite these elevated risk behaviors, HIV testing and PrEP campaigns have largely ignored trans men and nonbinary individuals assigned female at birth, which have left these populations with lower utilization of HIV prevention services, lower risk perceptions for HIV, and less interest in taking PrEP [48]. In our study, nonbinary participants, in particular, were less engaged in care compared to trans individuals.

A pillar of many HIV prevention campaigns is the push to get those at most risk for HIV infection into medical care and HIV testing [47, 92]. Most participants in this sample had ever been tested for HIV and had a regular source of care, but only half were retained in routine medical care. Participants who had seen a medical doctor within the last 6 months were more likely to report lifetime PrEP use than those who were not recently engaged in medical care. Lower medical engagement has been associated with PrEP use among Black and Latina trans women [41]. Lower rates of HIV testing, linkage and retention in care among nonbinary participants of both sexes assigned at birth likely explained why no differences in healthcare utilization were observed based on sex assigned at birth in the unstratified model. Although no direct effects on PrEP uptake were observed by region, the indirect relationship between region and PrEP uptake mediated by healthcare utilization suggests the importance of reducing barriers to care for TGEYYA in the South as well as for nonbinary youth across the country [29].

Several limitations should be considered in light of these findings. First, we were unable to assess willingness and intentions to start PrEP because the PrEP Motivational Cascade was not used in the CARES study, a useful measure which might provide additional psychological insight into motivations for taking PrEP, while also considering the impact of structural and behavioral barriers to initiating and persisting on PrEP [51]. Second, PrEP uptake was assessed based on lifetime use due to low current use, so the lifetime outcome might not align with behavioral risk data collected in the last 3 months. Third, differences in the recruitment timing and procedures of the two studies (i.e., mostly in-person recruitment before the COVID-19 pandemic for CARES vs. mostly online recruitment during the COVID-19 pandemic for TechStep) may have led to different characteristics of each sample or different patterns of responses. Given that most TechStep participants enrolled during the COVID-19 pandemic, it is possible that unmet structural needs and fewer behavioral risks reported by TechStep participants were reflective of widespread trends of increased social isolation and staying at home across the U.S. rather than the specific life circumstances of the TechStep participants [99]. Fourth, we were unable to account for partner HIV status due to incongruity in the questions asked between studies, which is an important factor for determining PrEP eligibility and perceived need for PrEP. Nonetheless, the high prevalence of recent condomless anal and vaginal sex and having multiple sex partners suggests that most participants met CDC PrEP indication criteria even in the absence of information on partner HIV status [63].

Additionally, we limited the number of parameters in the GSEM models to maximize power and ensure convergence and interpretability, so some pathways, covariates, and moderators were excluded from the final models. As such, omitted variable bias could not be entirely ruled out. First, the pathway between no perceived need for PrEP and healthcare utilization was not included in the GSEM models because these variables were not associated in the unadjusted bivariate model (OR: 0.899, 95% CI: 0.626–1.291, p = 0.565) nor in the adjusted multivariable model when controlling for other covariates (AOR: 1.260, 95% CI: 0.813–1.953, p = 0.301). This lack of association could be due to the healthcare utilization variable being too general (i.e., linkage to a usual source of care and doctor visits for any reason), whereas a more specific measure related to utilization of HIV prevention services would be expected to correlate with perceived need for PrEP. Second, age was not included as a covariate in the GSEM models because age was not associated with PrEP uptake or the mediators. No observed correlations between age and any of the outcomes of interest for this analysis might be due to the narrow age range of adolescents in our sample with less than 10 years between the youngest and oldest study participants. The majority of participants (72%) were between 20 and 24 years old at enrollment and less than 10% were below 18 years old. Future studies with individuals at different life course stages should evaluate potential age disparities in PrEP use among TGEYYA, as well as disparities by other demographic characteristics such as race/ethnicity and educational attainment. Finally, the relationships between gender identity and mediators could have been conditional on other factors. Methods to incorporate interactions into GSEM were limited by the sample size and underscore additional directions for future research. However, the consistent results of our sensitivity analyses modeling the outcome and mediators separately using regression models with all covariates suggest that the inclusion of additional parameters in the GSEM models likely would not change the key findings of this analysis.

Despite these limitations, our study provides important insights on mechanisms of PrEP uptake among TGEYYA and multiple points of intervention to reduce PrEP disparities and improve uptake across the gender identity spectrum. This ATN cross-network analysis included a diverse sample of TGEYYA with multiple intersecting gender, sexual, and racial/ethnic minority identities, as well as a broad geographic reach from six major cities across the West, South and Northeast regions of the U.S. In addition to characterizing key drivers of PrEP use among TGEYYA across the country, our findings also highlight the need to reduce barriers to care for trans youth and young adults in the South and among nonbinary youth and young adults throughout the U.S. who remain underserved by existing HIV prevention efforts.

## Data Availability

This analysis uses data from two studies, CARES (ATN 149) and TechStep (ATN 160). Both of these studies will have data available in NIH DASH repository per the funders’ requirements.

## References

[1] Mehringer J, Dowshen NL. Sexual and reproductive health considerations among transgender and gender-expansive youth. Curr Probl Pediatr Adolesc Health Care. 2019;49(9):100684. 31735693 10.1016/j.cppeds.2019.100684

[2] Baral SD, et al. Worldwide burden of HIV in transgender women: a systematic review and meta-analysis. Lancet Infect Dis. 2013;13(3):214–22. 23260128 10.1016/S1473-3099(12)70315-8

[3] Poteat, T., et al., *Global epidemiology of HIV infection and related syndemics affecting transgender people.* Journal of acquired immune deficiency syndromes (1999), 2016. **72**(Suppl 3): p. S210. 10.1097/QAI.0000000000001087 PMC4969059 27429185

[4] Becasen JS, et al. Estimating the prevalence of HIV and sexual behaviors among the US transgender population: a systematic review and meta-analysis, 2006–2017. Am J Public Health. 2019;109(1):e1–8. 30496000 10.2105/AJPH.2018.304727 PMC6301428

[5] Linley, L., et al., *Estimated HIV incidence and prevalence in the United States, 2014–2018.* 2020.

[6] Todd K, et al. Demographic and behavioral profiles of nonbinary and binary transgender youth. Transgend Health. 2019;4(1):254–61. 31641692 10.1089/trgh.2018.0068 PMC6802728

[7] Del Río-González AM, et al. Global scoping review of HIV prevention research with transgender people: TRANSCENDING from trans-subsumed to trans-centred research. J Int AIDS Soc. 2021;24(9): e25786. 34473421 10.1002/jia2.25786 PMC8412127

[8] Edmiston EK, et al. Opportunities and gaps in primary care preventative health services for transgender patients: a systemic review. Transgend Health. 2016;1(1):216–30. 28861536 10.1089/trgh.2016.0019 PMC5367473

[9] Feldman JL, et al. Health and health care access in the US transgender population health (TransPop) survey. Andrology. 2021;9(6):1707–18. 34080788 10.1111/andr.13052 PMC8613303

[10] Fisher CB, et al. Perceived barriers to HIV prevention services for transgender youth. LGBT Health. 2018;5(6):350–8. 30070960 10.1089/lgbt.2017.0098 PMC6145040

[11] Gianella S, et al. The importance of human immunodeficiency virus research for transgender and gender-nonbinary individuals. Clin Infect Dis. 2018;66(9):1460–6. 29126186 10.1093/cid/cix990 PMC5905620

[12] Herbst JH, et al. Estimating HIV prevalence and risk behaviors of transgender persons in the united states: a systematic review. AIDS Behav. 2008;12(1):1–17. 17694429 10.1007/s10461-007-9299-3

[13] Golub SA, et al. 1999 High rates of PrEP eligibility but low rates of PrEP access among a national sample of transmasculine individuals. J Acquir Immun Defic Syndr. 2019;82(1):e1–7. 10.1097/QAI.0000000000002116 PMC6692190 31232834

[14] Horvath KJ, et al. Underutilization of pre-exposure prophylaxis services among transgender and nonbinary youth: findings from project moxie and techstep. Transgend Health. 2019;4(1):217–21. 31592151 10.1089/trgh.2019.0027 PMC6778317

[15] Zamantakis A, et al. Determinants of pre-exposure prophylaxis (PrEP) implementation in transgender populations: a qualitative scoping review. AIDS Behav. 2023;27(5):1600–18. 36520334 10.1007/s10461-022-03943-8 PMC9753072

[16] Theodore DA, et al. Pre-exposure prophylaxis use among predominantly african american and hispanic women at risk for HIV acquisition in New York City. J Assoc Nurses AIDS Care. 2020;31(1):110–4. 31789687 10.1097/JNC.0000000000000147 PMC7380512

[17] Westmoreland DA, et al. Individual and partner characteristics associated with intentions to use PrEP among partnered men, trans men, and trans women in sero-concordant and-discordant relationships in the United States. AIDS Educ Prev. 2020;32(5):367–77. 33112677 10.1521/aeap.2020.32.5.367 PMC7597370

[18] Klein A, Golub SA. Increasing access to pre-exposure prophylaxis among transgender women and transfeminine nonbinary individuals. AIDS Patient Care STDS. 2019;33(6):262–9. 31166785 10.1089/apc.2019.0049

[19] MacCarthy S, et al. Current research gaps: a global systematic review of HIV and sexually transmissible infections among transgender populations. Sexual health. 2017;14(5):456–68. 29216970 10.1071/SH17096 PMC6993920

[20] Poteat T, et al. HIV prevention among transgender populations: knowledge gaps and evidence for action. Curr HIV/AIDS Rep. 2017;14(4):141–52. 28752285 10.1007/s11904-017-0360-1 PMC5896563

[21] Herman, J.L., A.R. Flores, and K.K. O'Neill, *How many adults and youth identify as transgender in the United States?* 2022.

[22] James SE, et al. The report of the 2015 US transgender survey. Washington, DC: National Center for Transgender Equality. National Center for Transgender Equality; 2016.

[23] Flores, A.R., T.N. Brown, and J. Herman, *Race and ethnicity of adults who identify as transgender in the United States*. 2016: Williams Institute, UCLA School of Law Los Angeles, CA.

[24] Gates, G.J., *How many people are lesbian, gay, bisexual and transgender?* 2011.

[25] Storholm ED, Huang W, Ogunbajo A, Horvath KJ, Reback CJ, Blumenthal J, Moore DJ, Flynn RP, Bolan RK, Corado KC, Morris SR. Gender-based violence and post-traumatic stress disorder symptoms predict HIV PrEP uptake and persistence failure among transgender and non-binary persons participating in a PrEP demonstration project in southern California. AIDS Behav. 2023;27(2):745–59. 36053404 10.1007/s10461-022-03807-1 PMC9908815

[26] Stotzer RL. Violence against transgender people: a review of United States data. Aggress Violent Beh. 2009;14(3):170–9.

[27] Fletcher JB, Kisler KA, Reback CJ. Housing status and HIV risk behaviors among transgender women in Los Angeles. Arch Sex Behav. 2014;43(8):1651–61. 25190499 10.1007/s10508-014-0368-1 PMC4214608

[28] Grant, J.M., et al., *Transgender Discrimination Survey.* National Center for Transgender Equality and National Gay and Lesbian Task Force: Washington, DC, USA, 2011.

[29] Harrison SE, et al. “Do I want PrEP or do I want a roof?”: social determinants of health and HIV prevention in the southern United States. AIDS Care. 2022. 10.1080/09540121.2022.2029816. 35109734 10.1080/09540121.2022.2029816 PMC9343473

[30] Jaffee KD, Shires DA, Stroumsa D. Discrimination and delayed health care among transgender women and men. Med Care. 2016;54(11):1010–6. 27314263 10.1097/MLR.0000000000000583

[31] Bauer GR, et al. High heterogeneity of HIV-related sexual risk among transgender people in Ontario, Canada: a province-wide respondent-driven sampling survey. BMC Public Health. 2012;12(1):1–12. 22520027 10.1186/1471-2458-12-292 PMC3424163

[32] Aggarwal NK, et al. Health and health care access barriers among transgender women engaged in sex work: a synthesis of US-based studies published 2005–2019. LGBT health. 2021;8(1):11–25. 33297834 10.1089/lgbt.2019.0243

[33] Fehrenbacher AE, et al. Social networks and exchange sex among transgender women. J Sex Res. 2021;58(6):743–53. 33779427 10.1080/00224499.2021.1892575 PMC8273090

[34] Poteat T, et al. HIV risk and preventive interventions in transgender women sex workers. The Lancet. 2015;385(9964):274–86. 10.1016/S0140-6736(14)60833-3 PMC4320978 25059941

[35] Hughto JMW, et al. A multisite, longitudinal study of risk factors for incarceration and impact on mental health and substance use among young transgender women in the USA. J Public Health. 2019;41(1):100–9. 10.1093/pubmed/fdy031 PMC6490767 29474682

[36] Legal, L., *Transgender incarcerated people in crisis.* Know Your Rights, nd.

[37] Daum CW. The war on solicitation and intersectional subjection: Quality-of-life policing as a tool to control transgender populations. New Polit Sci. 2015;37(4):562–81.

[38] Fitzgerald, E., et al., *Meaningful work: Transgender experiences in the sex trade*. 2015: National Center for Transgender Equality.

[39] Day JK, et al. Transgender youth substance use disparities: results from a population-based sample. J Adolesc Health. 2017;61(6):729–35. 28942238 10.1016/j.jadohealth.2017.06.024 PMC6802742

[40] Parrish KC, Johnson HZ, Williams SL. PrEP navigation continuum among men who have sex with men, trans women, and people with alternative gender identities in three California counties. Eval Program Plann. 2022;90: 101998. 34544606 10.1016/j.evalprogplan.2021.101998

[41] Nieto O, et al. Barriers and motivators to pre-exposure prophylaxis uptake among Black and Latina transgender women in los angeles: perspectives of current PrEP users. AIDS Care. 2021;33(2):244–52. 32449399 10.1080/09540121.2020.1769835 PMC7680715

[42] Reback CJ, et al. A promising PrEP navigation intervention for transgender women and men who have sex with men experiencing multiple syndemic health disparities. J Community Health. 2019;44(6):1193–203. 31317438 10.1007/s10900-019-00705-x PMC6859945

[43] Watson RJ, et al. PrEP stigma and logistical barriers remain significant challenges in curtailing HIV transmission among black and hispanic/latinx cisgender sexual minority men and transgender women in the US. AIDS Care. 2022. 10.1080/09540121.2022.2098908. 35848490 10.1080/09540121.2022.2098908 PMC9842805

[44] Kachen A, Pharr JR. Health care access and utilization by transgender populations: a United States transgender survey study. Transgender Health. 2020;5(3):141–8. 33644308 10.1089/trgh.2020.0017 PMC7906231

[45] Kattari SK, et al. One size does not fit all: differential transgender health experiences. Soc Work Health Care. 2019;58(9):899–917. 31618117 10.1080/00981389.2019.1677279

[46] McNairy ML, El-Sadr WM. A paradigm shift: focus on the HIV prevention continuum. Clin Infect Dis. 2014. 10.1093/cid/ciu251. 24926026 10.1093/cid/ciu251 PMC4141493

[47] Brooks RA, et al. Delivering PrEP to adults with “low” or “no” HIV risk and youth: experiences and perspectives of PrEP providers. Cult Health Sex. 2022. 10.1080/13691058.2020.1817560. 10.1080/13691058.2020.1817560 PMC8007677 32996431

[48] Dang M, et al. Barriers and facilitators to HIV pre-exposure prophylaxis uptake, adherence, and persistence among transgender populations in the united states: a systematic review. AIDS Patient Care STDS. 2022;36(6):236–48. 35687813 10.1089/apc.2021.0236 PMC9242706

[49] Knox J, et al. Assessing the information-motivation-behavioral skills model to predict pre-exposure prophylaxis adherence among black men who have sex with men and transgender women in a community setting in New York City. AIDS Behav. 2022;26(7):2494–502. 35098392 10.1007/s10461-022-03588-7 PMC9167713

[50] Golub SA, et al. Predictors of PrEP uptake among patients with equivalent access. AIDS Behav. 2019;23(7):1917–24. 30600456 10.1007/s10461-018-2376-y PMC6571035

[51] Parsons JT, et al. Uptake of HIV pre-exposure prophylaxis (PrEP) in a national cohort of gay and bisexual men in the United States: the motivational PrEP cascade. J Acquired Immu Defic Syndr. 1999;74(3):285. 10.1097/QAI.0000000000001251 PMC5315535 28187084

[52] CDC, *HIV infection, risk, prevention and testing behaviors among transgender women–National HIV behavioral surveillance, 7 US cities, 2019–2020.* HIV Surveill Special Rep, 2021. **27**: p. 15.

[53] Reisner SL, Murchison GR. A global research synthesis of HIV and STI biobehavioural risks in female-to-male transgender adults. Glob Public Health. 2016;11(7–8):866–87. 26785800 10.1080/17441692.2015.1134613 PMC4993101

[54] Radix AE, et al. HIV prevalence among transmasculine individuals at a New York City community health centre: a cross-sectional study. J Int AIDS Soc. 2022. 10.1002/jia2.25981. 36225145 10.1002/jia2.25981 PMC9557011

[55] Reisner SL, et al. Syndemics and gender affirmation: HIV sexual risk in female-to-male trans masculine adults reporting sexual contact with cisgender males. Int J STD AIDS. 2016;27(11):955–66. 26384946 10.1177/0956462415602418 PMC4798921

[56] Reisner SL, et al. Sexual risk behaviors and psychosocial health concerns of female-to-male transgender men screening for STDs at an urban community health center. AIDS Care. 2014;26(7):857–64. 24206043 10.1080/09540121.2013.855701 PMC4634528

[57] Tree-McGrath CAF, et al. Sexuality and gender affirmation in transgender men who have sex with cisgender men. Int J Trans. 2018;19(4):389–400.

[58] Rowniak, S., et al., *Transmen: the HIV risk of gay identity.* 2011. 10.1521/aeap.2011.23.6.508 22201235

[59] Deutsch MB. Pre-exposure prophylaxis in trans populations: providing gender-affirming prevention for trans people at high risk of acquiring HIV. LGBT health. 2018;5(7):387–90. 30272493 10.1089/lgbt.2018.0086

[60] Deutsch MB, et al. HIV pre-exposure prophylaxis in transgender women: a subgroup analysis of the iPrEx trial. Lancet HIV. 2015;2(12):e512–9. 26614965 10.1016/S2352-3018(15)00206-4 PMC5111857

[61] Escudero DJ, et al. Inclusion of trans women in pre-exposure prophylaxis trials: a review. AIDS Care. 2015;27(5):637–41. 25430940 10.1080/09540121.2014.986051 PMC4336598

[62] Carneiro PB, et al. Demographic, clinical guideline criteria, Medicaid expansion and state of residency: a multilevel analysis of PrEP use on a large US sample. BMJ Open. 2022;12(2):e055487. 35110323 10.1136/bmjopen-2021-055487 PMC8811583

[63] Mayeux JJ, Ng YC. Preexposure prophylaxis for HIV prevention: a highlight of the updated clinical practice guideline. Nurse Pract. 2022;47(9):44–7. 36006820 10.1097/01.NPR.0000855308.51321.39

[64] Downing J, Yee K, Sevelius JM. PrEP use and adherence among transgender patients. AIDS Behav. 2022;26(4):1251–9. 34643827 10.1007/s10461-021-03482-8 PMC9351441

[65] Shannon CL, et al. Sexually transmitted infection positivity among adolescents with or at high-risk for human immunodeficiency virus-infection in Los Angeles and New Orleans. Sex Transm Dis. 2019;46(11):737. 31453926 10.1097/OLQ.0000000000001056 PMC6812613

[66] Morris, E., *Characteristics associated with pre-exposure prophylaxis discussion and use among transgender women without HIV infection—National HIV Behavioral Surveillance Among Transgender Women, seven urban areas, United States, 2019–2020.* MMWR supplements, 2024. **73**. 10.15585/mmwr.su7301a2 PMC10826686 38261546

[67] Brooks RA, et al. Experiences of pre-exposure prophylaxis stigma, social support, and information dissemination among Black and Latina transgender women who are using pre-exposure prophylaxis. Transgender health. 2019;4(1):188–96. 31482134 10.1089/trgh.2019.0014 PMC6716188

[68] Seelman KL, Poteat T. Strategies used by transmasculine and non-binary adults assigned female at birth to resist transgender stigma in healthcare. Int J Trans Health. 2020;21(3):350–65. 10.1080/26895269.2020.1781017 PMC8726602 34993514

[69] Rodriguez A, et al. Awareness and utilization of pre-exposure prophylaxis and HIV prevention services among transgender and non-binary adolescent and young adults. Front Reprod Health. 2024;5:1150370. 38318604 10.3389/frph.2023.1150370 PMC10839107

[70] Hostetter CR, et al. “We are doing the absolute most that we can, and no one is listening”: barriers and facilitators to health literacy within transgender and nonbinary communities. Int J Environ Res Public Health. 2022;19(3):1229. 35162254 10.3390/ijerph19031229 PMC8834767

[71] Burchell D, et al. ‘I don’t want to have to teach every medical provider’: barriers to care among non-binary people in the Canadian healthcare system. Cult Health Sex. 2024;26(1):61–76. 37173293 10.1080/13691058.2023.2185685

[72] Swendeman D, et al. Text-messaging, online peer support group, and coaching strategies to optimize the HIV prevention continuum for youth: protocol for a randomized controlled trial. JMIR Res Protoc. 2019;8(8): e11165. 31400109 10.2196/11165 PMC6707028

[73] Ocasio MA, et al. Engaging sexual and gender minority youth in hiv interventions through gay dating apps: recruitment protocol. JMIR Res Protoc. 2021;10(6):e28864. 34156342 10.2196/28864 PMC8277323

[74] Reback CJ, et al. Technology-based stepped care to stem transgender adolescent risk transmission: protocol for a randomized controlled trial (TechStep). JMIR Res Protoc. 2020;9(8):e18326. 32788149 10.2196/18326 PMC7458064

[75] Link BG, Phelan J. Social conditions as fundamental causes of disease. J Health Soc Behav. 1995. 10.2307/2626958. 7560851

[76] Felsher M, et al. “I don’t need prep right now”: a qualitative exploration of the barriers to prep care engagement through the application of the health belief model. AIDS Educ Prev. 2018;30(5):369–81. 30332306 10.1521/aeap.2018.30.5.369 PMC8558876

[77] Reback CJ, et al. Text messaging to improve linkage, retention, and health outcomes among hiv-positive young transgender women: protocol for a randomized controlled trial (Text Me, Girl!). JMIR Res Protoc. 2019;8(7): e12837. 31359867 10.2196/12837 PMC6690158

[78] Elopre L, et al. Missed prevention opportunities: why young, black MSM with recent HIV diagnosis did not access HIV pre-exposure prophylaxis services. AIDS Behav. 2021;25:1464–73. 32749626 10.1007/s10461-020-02985-0 PMC7858694

[79] R Core Team. *R: A language and environment for statistical computing*. 2021; Available from: https://www.R-project.org/.

[80] CDC. *Deciding to Take PrEP*. 2021 July 6, 2022 [cited 2022; Available from: https://www.cdc.gov/hiv/basics/prep/prep-decision.html.

[81] CDC. *PrEP for HIV Prevention in the U.S.* . 2021; Available from: https://www.cdc.gov/nchhstp/newsroom/fact-sheets/hiv/PrEP-for-hiv-prevention-in-the-US-factsheet.html.

[82] CDC. *HIV Infection, Risk, Prevention, and Testing Behaviors Among Transgender Women—National HIV Behavioral Surveillance, 7 U.S. Cities, 2019–2020. HIV*

[83] *Surveillance Special Report 27*. 2021 [cited 2022; Available from: https://www.cdc.gov/hiv/pdf/library/reports/surveillance/cdc-hiv-surveillance-special-report-number-27.pdf.

[84] Sullivan PS, et al. Trends in the use of oral emtricitabine/tenofovir disoproxil fumarate for pre-exposure prophylaxis against HIV infection, United States, 2012–2017. Ann Epidemiol. 2018;28(12):833–40. 30037634 10.1016/j.annepidem.2018.06.009 PMC6286244

[85] Kimball AA, et al. HIV preexposure prophylaxis provision among adolescents: 2018 to 2021. Pediatrics. 2023;152(5):e2023062599. 37899721 10.1542/peds.2023-062599

[86] Shorrock F, et al. Dismantling barriers and transforming the future of pre-exposure prophylaxis uptake in young black and latinx sexual minority men and transgender women. AIDS Patient Care STDS. 2022;36(5):194–203. 35507322 10.1089/apc.2021.0222 PMC9125574

[87] Huebner DM, Mustanski B. Navigating the long road forward for maximizing PrEP impact among adolescent men who have sex with men. Arch Sex Behav. 2020;49(1):211–6. 31667642 10.1007/s10508-019-1454-1 PMC7665846

[88] Fisher CB, et al. Facilitators and barriers to participation in PrEP HIV prevention trials involving transgender male and female adolescents and emerging adults. AIDS Education Prevent. 2017;29(3):205. 10.1521/aeap.2017.29.3.205 PMC5768197 28650227

[89] Hines DD, Ryan M. It’s not just about condoms and sex: using syndemic theory to examine social risks of HIV among transgender women. In: Understanding the HIV/AIDS Epidemic in the United States. Springer; 2016. p. 99–130.

[90] King WM, et al. Structural needs, substance use, and mental health among transgender and nonbinary young adults in the san francisco bay area: findings from the phoenix study. J Urban Health. 2023. 10.1007/s11524-022-00700-z. 36595118 10.1007/s11524-022-00700-z PMC9918689

[91] Eaton LA, et al. A multi-us city assessment of awareness and uptake of pre-exposure prophylaxis (PrEP) for HIV prevention among black men and transgender women who have sex with men. Prev Sci. 2017;18:505–16. 28101813 10.1007/s11121-017-0756-6 PMC5926200

[92] Kuhns LM, et al. Correlates of PrEP indication in a multi-site cohort of young HIV-uninfected transgender women. AIDS Behav. 2016;20(7):1470–7. 26336946 10.1007/s10461-015-1182-z PMC4777686

[93] Reback CJ, et al. Recruiting, linking, and retaining high-risk transgender women into HIV prevention and care services: an overview of barriers, strategies, and lessons learned. International Journal of Transgenderism. 2015;16(4):209–21. 27110227 10.1080/15532739.2015.1081085 PMC4838285

[94] Nieto O, et al. PrEP discontinuation among Latino/a and Black MSM and transgender women: a need for PrEP support services. PLoS ONE. 2020;15(11): e0241340. 33151997 10.1371/journal.pone.0241340 PMC7644013

[95] Ayangeakaa SD, Kerr J, Combs R, Harris L, Sears J, Parker K, Sterrett-Hong E. Understanding influences on intention to use pre-exposure prophylaxis (PrEP) among african american young adults. J Racial Ethn Health Disparities. 2023;10(2):899–910. 35290648 10.1007/s40615-022-01278-7

[96] Fitch C, et al. Individual and structural-level Correlates of Pre-exposure Prophylaxis (PrEP) lifetime and current use in a nationwide sample of young sexual and gender minorities. AIDS Behav. 2022;26:3365. 35429311 10.1007/s10461-022-03656-y PMC9474722

[97] Zarwell M, John SA, Westmoreland D, Mirzayi C, Pantalone DW, Golub S, Nash D, Grov C. PrEP uptake and discontinuation among a U.S. national sample of transgender men and women. AIDS Behav. 2021;25:1063–71. 33057893 10.1007/s10461-020-03064-0 PMC7979462

[98] Bauer GR, et al. Sexual health of trans men who are gay, bisexual, or who have sex with men: results from Ontario. Canada Int J Trans. 2013;14(2):66–74. 10.1080/15532739.2013.791650 PMC4059421 24971043

[99] Antebi-Gruszka N, et al. Sociodemographic and behavioural factors associated with testing for HIV and STIs in a US nationwide sample of transgender men who have sex with men. Sex Trans Infect. 2020;96(6):422–7. 10.1136/sextrans-2020-054474 PMC7653680 32605930

[100] D’Angelo AB, et al. Health and access to gender-affirming care during COVID-19: experiences of transmasculine individuals and men assigned female sex at birth. Am J Mens Health. 2021;15(6):15579883211062680. 34861796 10.1177/15579883211062681 PMC8646200

